# Does your discussion realize its potential?

**DOI:** 10.1007/s40037-017-0377-6

**Published:** 2017-09-04

**Authors:** Lorelei Lingard

**Affiliations:** Schulich School of Medicine & Dentistry, Health Sciences Addition, Western University, London, Ontario Canada

In the Writer’s Craft section we offer simple tips to improve your writing in one of three areas: Energy, Clarity and Persuasiveness. Each entry focuses on a key writing feature or strategy, illustrates how it commonly goes wrong, teaches the grammatical underpinnings necessary to understand it and offers suggestions to wield it effectively. We encourage readers to share comments on or suggestions for this section on Twitter, using the hashtag: #how’syourwriting?

A few months ago one of my research teams got a ‘Major Revision’ response from a journal after our manuscript was reviewed. The main critique was that ‘the Discussion does not realize the potential of the Introduction’. That critique sums up beautifully the purpose of a Discussion section: it must bring to fruition the story that the Introduction started. But how can we ensure that our Discussions succeed in this aim?

Together, the Introduction and Discussion sections of a manuscript *tell the story*, while the Methods and Results sections *report the study *[1]. For a manuscript to tell a coherent story, its Discussion section ought to provide the *so what?,* the climax of the work. Too often, however, we fall short. We settle for Discussions that merely summarize results, confess limitations, and suggest future research. This tendency reflects a well-worn ‘formula’ but it fails to create any narrative arc in the story, leaving much room for improvement and artistry. This Writer’s Craft instalment offers two strategies writers can use to improve their Discussion sections: 1) Think of your ideas as characters in a drama, and carefully consider how to shape their story arcs, and 2) Create a recognizable storyline linking your Introduction and Discussion.

## Who are your main characters?

A drama metaphor can help you to identify, position and develop the characters in your research story. Think of the Introduction of your paper as the opening Act of a play, and each idea you introduce as a character. How many characters are you bringing on stage? Is the main character clearly indicated? Imagine a manuscript about assessing student professionalism that introduces the following characters in the opening paragraphs: professionalism, ethics, assessment, clerkship, dilemmas, competency, observation, feedback and role modelling. This stage is overly full! Pare down the characters, introduce them carefully and ensure that the main character(s) stand out clearly and supporting characters are put in their place. Now, think of the Discussion as Act III, the climax of the story. Which of your supporting characters must return? What will happen to the main character(s) so that they develop? Have you introduced a new main character in the Discussion, or killed off the main character? Both of these are abrupt departures from the conventional storylines in a research manuscript – not impossible, but only to be done purposefully and carefully.

## What’s your storyline?

As the drama metaphor emphasizes, the Introduction and Discussion are in partnership. You can think of their relationship as a storyline that influences when characters appear and how they develop. In my experience, three storylines recur in health professions education research manuscripts: *Coming full circle, Deep exploration* and *Surprise insight*. Learning to recognize these storylines can help writers to assess the conventions of the journal they’re targeting and the affordances of particular storylines for their manuscript.

In a *Coming full circle* storyline, each idea/character presented in the Introduction returns in the Discussion. No new characters are introduced in the Discussion, as all relevant concepts and literatures have been set out in the Introduction and are revisited methodically in the Discussion. This storyline is signalled by few or no new keywords or references in the Discussion section of the paper. It is important to recognize that this storyline is not a signal that no new results emerged in the study! Results are study, not story, and they do not necessarily dictate the storyline structure of the Introduction and Discussion sections. *Coming full circle* is a common structure in quantitative and experimental research manuscripts, where research designs focus on a defined research question. For instance, in a paper describing a randomized controlled trial of two resident duty hours models in critical care, the Introduction presented the main characters/issues of physician fatigue, patient safety, duty hours and care continuity, and the Discussion revisited each of these while interpreting study results in light of the literature [2]. The difference between *Coming full circle* and the tired Discussion ‘formula’ is the building of this purposeful storyline between the Introduction and Discussion.

In a *Deep exploration* storyline, the Discussion selectively explores a subset of ideas from the Introduction. In this storyline, the main character in the Discussion is not new; it will have been presented in the Introduction. However, new supporting characters may be brought on stage in the Discussion as part of the deep exploration of this character’s world. This will be signalled by new keywords and references in the Discussion section. In *Deep exploration* storylines, the first paragraph of the Discussion will often provide a brief review of the full company of ideas/characters presented in the Introduction, before diving into one or two in more extensive detail. *Deep exploration* is a common storyline in social sciences research writing generally and health professions education manuscripts specifically. For instance, in a paper describing the development of an observation tool to measure team communication, the Introduction presented the main characters of team communication, communication failure, improvement initiatives, and performance measurement; the Discussion, however, focused mostly on performance measurement, detailing in particular the trade-offs between reliability and authenticity [3].

In a *Surprise insight* storyline, the Discussion introduces new ideas or main characters that were not presented in the Introduction. Sometimes this new idea will be briefly foreshadowed in the Introduction – picture a hooded character who slowly crosses the stage but does not speak – and other times it emerges as entirely new in the Discussion. *Surprise insight* storylines include many new references in the Discussion, often from literatures not broached in the Introduction as the writer elaborates the world of this new character. *Surprise insight* storylines are rare, but more likely in qualitative and constructivist research manuscripts, where the research approach invites emergent twists and turns not imagined at the outset of the study. For example, in a paper from our healthcare teamwork research, the Introduction spotlighted the main characters of inter-professional care, collaboration and leadership. The Discussion summarized the key finding of a double bind for physicians navigating the competing values of leadership and collaboration, and then explicitly introduced a new suite of characters that were necessary to understand this double bind: *“To explore this provocative explanation for our findings, we briefly consider three of the broader systems that support physicians’ privileged status: the education system, the health care delivery system, and the medical-legal system.” *[4, P. 1765] The bulk of the Discussion elaborated these new characters. As this example demonstrates, such explicit signposting – ‘this provocative explanation for our findings’ – is critical in a *Surprise insight* storyline, in order to minimize the chance that the reader may perceive a random detour. This example illustrates another important point: *Surprise insight* is not a reference to new or surprising results; it is a reference to a new character in the story told about the results. Many studies report new results, but this is not what is meant by a *Surprise insight *storyline. Results are study, not story. A *Surprise insight* storyline represents a decision to reveal some of the characters in the story in the Discussion rather than putting them all up front in the Introduction. In this case, it is the ‘provocative explanation’ of the results that is a surprise, not the results themselves.

Writers have some choice of storylines, but their choice must be guided by the conventions of the journal they wish to publish in. Consider questions such as: Will the journal expect symmetry of ideas between Introduction and Discussion? Does the journal allow new references in the Discussion? Will you need to foreshadow an idea in the Introduction or can it appear as new in the Discussion? Read papers in the journal to analyze the relationship between Introductions and Discussions, so that you can learn which storylines are common and discover adaptations of these structures.

Using the drama metaphor and storyline structure requires writers to shed the notion that the Introduction should represent what was known before the study was launched and the Discussion what is known after it is completed. A good story is rarely chronological, and good academic writers understand that readers do not need to come to understanding in the same stepwise manner that the researcher did. With this in mind, think about which characters should appear in your Intro/Act I, whether and how they need to reappear in your Discussion/Act III, or which new characters need introduction at this point to strengthen the narrative arc of the story.

Your research study – its methods and results – needs to be reported fully and accurately. Your research story, however, should be told as persuasively as possible. Knowledge translation is far more likely when we craft our research manuscripts to tell a compelling story about what we’ve learned.

## References

[1] Lingard L, Watling C (2016). It’s a Story, Not a Study: Writing an Effective Research Manuscript. Acad Med.

[2] Parshuram C, Friedrich J (2015). Amaral, et al. Patient safety, resident wellbeing and continuity in three resident duty schedules in ICU: a randomized trial. CMAJ.

[3] Lingard L, Regehr G, Espin S, Whyte S (2006). A theory-based instrument to evaluate team communication in the operating room: Balancing measurement authenticity and reliability. Qual Saf Health Care.

[4] Lingard L, Vanstone M, Durrant M (2012). Conflicting Messages: Examining the Dynamics of Leadership on Interprofessional Teams. Acad Med.

